# High-Dose Paraquat Induces Human Bronchial 16HBE Cell Death and Aggravates Acute Lung Intoxication in Mice by Regulating Keap1/p65/Nrf2 Signal Pathway

**DOI:** 10.1007/s10753-018-00956-1

**Published:** 2019-02-08

**Authors:** Jiexiong Yao, Jihua Zhang, Wenlin Tai, Shuhao Deng, Ting Li, Wenjuan Wu, Lin Pu, Du Fan, Wen Lei, Tao Zhang, Zhaoxing Dong

**Affiliations:** 1 Department of Internal Medicine Ward 5, Guangdong Provincial Corps Hospital of Chinese People’s Armed Police Forces, Guangzhou Medical University, Guangzhou, Guangdong 510507 People’s Republic of China; 2 Department of Pulmonary and Critical Care Medicine, The People Hospital of Yuxi City, Yuxi, China; 3 Department of Clinical Laboratory, Yunnan Molecular Diagnostic Center, The 2nd Affiliated Hospital of Kunming Medical University, Dianmian Road, Kunming, Yunnan China; 4 Department of Respiratory, The 2nd Affiliated Hospital of Kunming Medical University, Dianmian Road 374, Kunming, 650101 Yunnan China

**Keywords:** Paraquat, Lung fibrosis, Keap1, Nrf2, p65

## Abstract

Paraquat (PQ) intoxication seriously endangers human beings’ health, however, the underlying mechanisms are still unclear. Here we found that PQ inhibits human bronchial 16HBE cell proliferation and promotes cell apoptosis, necrosis as well as ROS generation in a dose dependent manner. Of note, low-dose PQ (50 μM) induces cell autophagy, increases Nrf2 as well as p65 levels and has little impacts on Keap1, while high-dose PQ (500 μM) inhibits autophagy, upregulates Keap1 as well as downregulates p65 and Nrf2. In addition, we verified that p65 overexpression increases Nrf2 and its downstream targets in 16HBE cells, which are reversed by synergistically knocking down Nrf2. Our further results showed that high-dose PQ’s effects on cell proliferation, apoptosis, ROS levels and autophagy are reversed by p65 overexpression. Besides, the protective effects of overexpressed p65 on high-dose PQ (500 μM) treated 16HBE cells are abrogated by synergistically knocking down Nrf2. *In vivo* experiments also showed that high-dose PQ promotes inflammatory cytokines secretion, lung fibrosis and cell apoptosis, inhibits cell proliferation in mice models by regulating Keap1/p65/Nrf2 signal pathway. Therefore, we concluded that high-dose PQ (500 μM) inhibits 16HBE cell proliferation and autophagy, promotes cell death and mice lung fibrosis by regulating Keap1/p65/Nrf2 signal pathway.

## INTRODUCTION

Paraquat (PQ) is one of the most common herbicides worldwide which is especially used in developing countries. PQ poisoning seriously endangered human being’s health, although it has been banned in most countries, it remains a commonly used agent causing death from self-poisoning with herbicide in developing countries [1, 2]. It has been reported that PQ poisoning causes human death by regulating DNA breakage [3], mitochondrial damage [4], cell apoptosis [5], and cell autophagy [6], but the detailed mechanisms of PQ-caused human death are still not fully delineated. Studies proved that accumulated PQ in lung tissues induces ROS generation [7] as well as inflammatory reactions [8], both of which play important roles in regulating lung fibrosis. The studies above indicated that ROS generation and inflammatory reactions might serve as therapeutic targets for PQ poisoning.

Recent study found that PQ treatment inhibits the viability of human neural progenitor cells by promoting ROS generation [9]. Besides, PQ induces hepatic injury in mice [10] and promotes mouse alveolar type II cell apoptosis by promoting ROS production. In addition to apoptosis, PQ promotes cell intoxication by regulating cell autophagy [11]. Autophagy has opposite effects on cells’ responses to environmental stress; on the one hand, autophagy prevents cells from apoptosis under environmental stresses [12]. On the other hand, autophagy-induced cell death has also been reported [11]. However, the role of autophagy in PQ-induced cell intoxication is still unclear. Of note, targeting paraquat-induced ROS generation has proved to be effective for the attenuation of lung fibrosis [13]. Furthermore, PQ-induced lung fibrosis is also closely related with inflammatory reactions, and anti-inflammatory agents have been reported to alleviate PQ-induced pulmonary injury in mice [14]. Therefore, PQ-induced ROS generation, cell autophagy, apoptosis, and inflammatory reactions might be pivotal for the pathogenesis of PQ-induced lung fibrosis, but the detailed mechanisms still need to be explored.

Kelch-like ECH-associated protein 1(Keap1)/p65/nuclear factor E2-related factor 2 (Nrf2) signal pathway is closely related with ROS generation. In normal cells, Keap1 binds to Nrf2 and activates its degradation processes [15]. However, Nrf2 is released from Keap1-Nrf2 complexes under the conditions with high ROS levels. Nrf2 binds to antioxidant response elements (AREs) in the nucleus and promotes the transcription of a wide variety of antioxidant genes, which helps to eliminate ROS levels and protect cells from ROS-induced cell death [16]. Keap1 physically associates with p65 [17], but it is still unclear whether p65 involves in the activation of Keap1/Nrf2 signal pathway. The activation of Keap1/Nrf2 signal pathway is also reported to regulate ROS-regulated cell autophagy [12] and apoptosis [18]. In addition, Nrf2 has also been reported to modulate inflammatory reactions [19].

Taken together, we hypothesized that PQ might induce ROS generation, cell apoptosis. and lung fibrosis by regulating Keap1/p65/Nrf2 signal pathway, and uncovering the underlying mechanisms might shed light on the discovery of therapeutic agents for PQ intoxication.

## MATERIALS AND METHODS

### Animals

Seven to 8 weeks male C57BL/6 mice (the total number was 20 and the weight of the mice ranged from 20 to 25 g) were purchased from Vital River Laboratory Animal Technology (Beijing, People’s Republic of China). All animal care and handling procedures were in accordance with the guidelines of the Institute for Laboratory Animal Research of the Second Affiliated Hospital of Kunming Medical University. The animal for research use was approved by the Animal Care and Use Committee of the Second Affiliated Hospital of Kunming Medical University. The animals were fed and maintained under the same conditions (temperature 23 ± 2 °C, humidity 55 ± 5%, 12:12 h light/dark cycle) in the Animal Research Center of Kunming Medical University. The mice were selected and divided into four groups and administered with 500 μM of PQ (Sigma-Aldrich, St. Louis, MO, USA) or the same volume of saline (control) by intraperitoneal injections. After 96 h, mice were sacrificed and the samples of lung tissues and peripheral blood were collected for the following studies.

### Detection of Tissue Morphology by Masson Staining

The Masson Staining Kit was purchased from Shanghai Bogoo Biotechnology Co., Ltd. (China). Mice lung tissues were collected and fixed in 4% paraformaldehyde for 24–48 h and embedded in paraffin wax for long-term preservation. Wax was then cut into 5 μM thicknesses. The paraffin-embedded sections were then de-paraffinized by xylene, and a series of ethanol were used to process and dehydrate the sections. The sections were then washed with phosphate buffer solution (PBS) three times, and the tissues were then stained with reagent A, B, and C sequentially according to the manufacturer’s protocol; optical microscope was used to observe tissue morphologies.

### Cell Culture and Cell Proliferation

16HBE cells were diluted into the density of 1 × 10^4^/ml and seeded into 96-well plates; cells were then cultured under the standard conditions (37 °C, 5% CO_2_) for 12 h. Different doses of PQ (50 μM, 150 μM, and 500 μM) were added into the wells (each assay has three repetitions) and co-cultured with cells for 12 h, 24 h, 48 h, 72 h, and 96 h respectively. Ten microliters of CCK-8 solution was added into each well and incubated in the plate for 1–4 h in the incubator following the manufacturer’s instructions of Cell Counting Kit-8 (MedChemExpress Co., Ltd., USA). Before reading the plate, the plate was gently mixed on an orbital shaker for 1 min; the optical density (OD) values were detected by a Gemini EM microplate reader (Molecular Devices, USA) at the absorbance of 450 nm. The OD values were used to evaluate cell proliferative abilities after being treated by different doses of PQ at various time points.

### Real-Time qPCR

After treating 16HBE cells and mice with different doses of PQ, TRIzol kit (Invitrogen, USA) was used to extract RNA from 16HBE cells or mice lung tissues following the manufacturer’s protocol. Reverse transcription PCR by iScript cDNA Synthesis Kit (Bio-Rad, Hercules, CA, USA) and real-time quantitative PCR by HiScript II Q Select RT SuperMix (Vazyme, China) were used to reverse and quantify relative GAPDH, Keap1, p65, Nrf2, IL-4, IL-6, IL-1β, and TNF-α expressions; the primers were designed and synthesized by Sangon Biotech Co., Ltd. (Shanghai, China); primers are listed in Table 1. Relative mRNA expression levels of genes were normalized by GADPH.

**Table 1:** Quantitative PCR Primers Used in the Study

Gene	Primer sequences (strand)
Keap1	Forward: 5′-CTACCTGGAGGCTTACAAC-3′
	Reverse: 5′-CATACCTCTCCACACTGTTG-3′
p65	Forward: 5′-AGATGGAGCCAGAGAACAAG-3′
	Reverse: 5′-TAAGAGCATAAGCCTCACATG-3′
Nrf2	Forward: 5′- CTCAGTCACCTGAAACTTCTG-3′
	Reverse: 5′-GCTGATACTGGGCTCAGCTATG-3′
IL-4	Forward: 5′-TGAGTGAGTGGTGGGGTCCTTAC-3′
	Reverse: 5′-CACTATGTTGCCTAGGCTCATCTC-3′
IL-6	Forward: 5′-CCAATTTCCAATGCTCTCCT-3′
	Reverse: 5′-ACCACAGTGAGGAATGTCCA-3′
IL-1β	Forward: 5′-ACAGTGGCAATGAGGATGAC-3′
	Reverse: 5′-GTTCATATGGACCAGACATC-3′
TNF-α	Forward: 5′-AGAAGGAAACAGACCACAGACC-3′
	Reverse: 5′-TCTGTAGTTGCTTCTCTCCCTC-3′
GAPDH	Forward: 5′-AGAAGGCTGGGGCTCATTTG-3′
	Reverse: 5′-AGGGGCCATCCACAGTCTTC-3′

### Western Blot

The samples of 16HBE cells and PQ-treated mice were collected, total protein was extracted using RIPA lysis buffer (Beyotime Biotechnology, Shanghai, China), and the protein concentration was determined with BCA Protein Assay Kit (Beyotime Biotechnology, Shanghai, China). Protein was then solubilized in 2× sample buffer; SDS-polyacrylamide gel electrophoresis was then performed to separate the targeted proteins. The proteins were then transferred to polyvinylidene difluoride (PVDF) membranes (Bio-Rad, Hercules, USA) and the membranes were incubated with 5% bovine serum albumin (BSA) for 60 min at the room temperature. The primary antibodies of anti-Keap1 (1:1000, #K2769, Sigma, USA), anti-p65 (1:1000, #P0068, Sigma, USA), anti-Nrf2 (1:500, #SAB4501984, Sigma, USA), anti-p21 (1:1000, #SAB4500065, Sigma, USA), anti-Cyclin A2 (1:1000, #C4710, Sigma, USA), anti-Cyclin D1 (1:1000, #C7464, Sigma, USA), anti-Bax (1:500, #B8429, Sigma, USA), anti-Bcl-2 (1:1000, #B3170, Sigma, USA), anti-Caspase 3 (1:500, #C8487, Sigma, USA), anti-LC-3 (1:1000, #8918, Sigma, USA), and anti-β-actin (1:1000, #A5441, Sigma, USA) were employed to be incubated with the membranes overnight at 4 °C. The anti-mouse IgG-peroxidase-conjugate secondary antibodies (Sigma, USA) were used to be incubated with the membrane for 2 h at the room temperature to combine to the primary antibodies. The bands were visualized by Enhanced Chemiluminescence Kit (Bio-Rad, USA) and ChemiDoc (Bio-Rad, USA); the expressions were quantified by ImageJ software. Each assay was repeated for at least 3 times to eliminate deviations.

### CRISPR-Cas9 Technology Was Used to Knockout Nrf2 in 16HBE Cells

CRISPR-Cas9 technology was conducted to knockout Nrf2 gene according to the protocol from Dr. Zhang F. The two sgRNAs targeting human Nrf2 were designed and constructed into pSgRNA (addgene#47108) using Bbs1 digestion (sgRNA sequences, F1:CAATTAAGGCATGGAATTCCCAT, R1: AAACCTATTCCCAGAGTCAGTCA; F2: CATTCCTCCAGGACCCTAGGATGCAGT, R2: AAACGTTCGAACTCTGAC-GGTA). In addition, the constructed plasmids and pCas9-GFP (addgene#44719) were transfected into 16HBE cells by Lipofectamine 3000 transfection reagent (#13778150, Invitrogen, USA) according to the manufacturer’s instructions. Approximately 1 × 10^6^ cells were transfected with 1 μg pCas9 plasmids, 1 μg pSgRNAs, and 0.2 μg PKG-puro plasmid with puromycin resistance gene. Puromycin (1.5 μg/ml) was then co-cultured with cells to select puromycin resistance cells. Total proteins were then extracted from the cells and Western Blot was used to verify the transfection efficiency of the plasmids.

### p65 Overexpression Plasmid Construction and Transfection

To overexpress cellular p65 expression levels, the coding sequence (GenBank S82307.1) for the human p65 gene was obtained from the total RNA of human HEK293T cells isolated by using TRIzolreragent (Takara Bio, Kusatsu, Japan) and then transcribed using iScript cDNA Synthesis Kit (Bio-Rad, Hercules, CA, USA). The sequences were then cloned into the pEGFP-N1 gene overexpression vectors, and the pEGFP-N1-p65 overexpression vectors were transfected into 16HBE cells by Lipofectamine 3000 transfection reagent (#13778150, Invitrogen, USA) according to the manufacturer’s instructions; the pEGFP-N1 empty vectors were used as the negative control. Western Blot was used to verify that the vectors were successfully transfected into the target cells.

### Flow Cytometry

16HBE cells treated with PQ were collected and prepared by GE Ficoll-Paque PLUS (GE, USA); cells were then treated with 10% DMSO and stored in − 80 °C. *Ex vivo* cellular staining for Annexin-V and PI was implemented by incubating cells with specific dyes (Thermo Fisher, USA) following the manufacturer’s instructions. Attune NxT Flow Cytometer (Thermo Fisher, USA) was used to collect the data of cell necrosis, early apoptosis, and late apoptosis. Each assay had at least 3 repetitions.

### Detection of ROS Levels

16HBE cells were treated with 500 μM of PQ for 0 h, 12 h, 24 h, and 48 h; L-012 dye was used to detect extracellular NADPH oxidase-derived superoxide. In brief, 16HBE cells were diluted into approximately 4–6 × 10^4^ cells/well into 96-well plates (Thermo, USA) in phenol-free DMEM medium (Sigma, USA) with L-012 at the concentration of 500 μM according to our preliminary experiments (data not shown) for 10 min and luminescence was detected by a Gemini EM microplate reader (Molecular Devices, USA) at the excitation wavelength of 488 nm and emission wavelength of 525 nm respectively. Cellular ROS levels were next measured by dihydroethidium (DHE) staining. Cells were washed with PBS twice and diluted; 10 μM of DHE (Invitrogen, USA) was selected according to our preliminary experiments (data not shown) to incubate with the cells for 30 min at 37 °C without light exposure. After incubation, cells were washed with PBS and DM500 fluorescence microscope (Leica, Germany) was employed to observe ROS productions. The fluorescence intensity was quantified and calculated by ImageJ software.

### Statistical Analysis

All the data collected in our experiments was showed as the mean ± standard deviation (SD), and the data was analyzed by SPSS 13.0 statistical software with one-way analysis of variance (ANOVA) for multiple groups and Student’s *t* test for two groups. *P* < 0.05 means statistical significance.

## RESULTS

### Cell Proliferation and Apoptosis of 16HBE Cells Treated with Different Doses of PQ

**Fig. 1:**
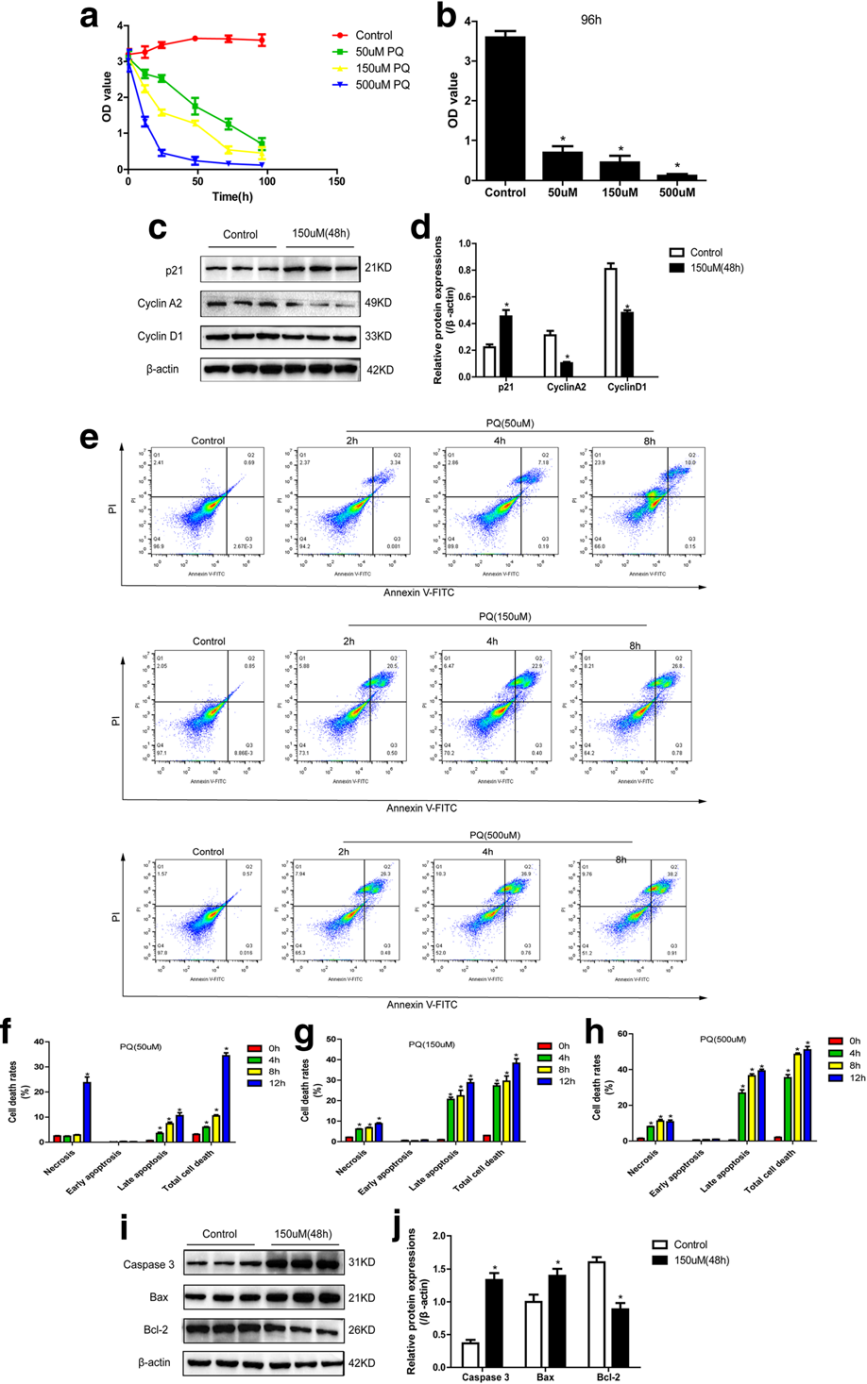
Different concentrations (50 μM, 150 μM, and 500 μM) of PQ’s influences on cell proliferation and apoptosis. **a** CCK-8 assay showed that PQ inhibits cell proliferative ability in time- and dose-dependent manners. **b** CCK-8 assay was used to detect different doses of PQ’s influences on cell proliferation at 96 h. **c** Cell cycle-associated proteins of 16HBE cells treated with PQ (150 μM) for 48 h were detected by Western Blot. **d** Quantification of cell cycle-associated proteins by ImageJ software. **e** Cell death rates were detected by FCM in 16HBE cells treated with different doses of PQ at 2 h, 4 h, and 8 h respectively. **f**, **g**, **h** Necrosis, early apoptosis, late apoptosis, and total cell death were counted according to the FCM results. **i** Apoptosis-associated proteins were detected by Western Blot in 16HBE cells treated with 150 μM PQ for 48 h. **j** Apoptosis-associated proteins were quantified by ImageJ software according to the Western Blot results. Each assay in the experiments had 3 repetitions (the data are presented as mean ± SD, “*” means statistical significance, *p* < 0.05).

Previous study proved that PQ induces cell intoxication in a dose-dependent manner [20]. In our study, 16HBE cells were treated with 50 μM, 150 μM, and 500 μM PQ for 12 h, 24 h, 48 h, and 72 h respectively; the results showed that the inhibiting effects of PQ on cell proliferation were positively correlated with PQ concentration and treating time (Fig. 1a–b), which were in line with the previous study [9]. The Western Blot results also showed that PQ (150 μM, 48 h) significantly increases p21 and decreases cyclin A2 as well as cyclin D1 in 16HBE cells (Fig. 1c–d), which indicated that cell cycle arrest is induced by PQ treatment. After treating cells with different concentrations of PQ (50 μM, 150 μM, and 500 μM) for 2 h, 4 h, and 8 h, respectively, the FCM results showed that PQ induces cell death and apoptosis in a dose-dependent manner (Fig. 1e). Of note, PQ specifically induces cell necrosis and late apoptosis instead of early apoptosis in 16HBE cells (Fig. 1f–h). In addition, pro-apoptotic proteins caspase 3 as well as Bax are increased, and anti-apoptotic protein Bcl-2 is decreased in 16HBE cells treated with 150 μM of PQ for 48 h compared with the control group (Fig. 1i–j).

### ROS Production in 16HBE Cells Treated with Different Doses of PQ

**Fig. 2:**
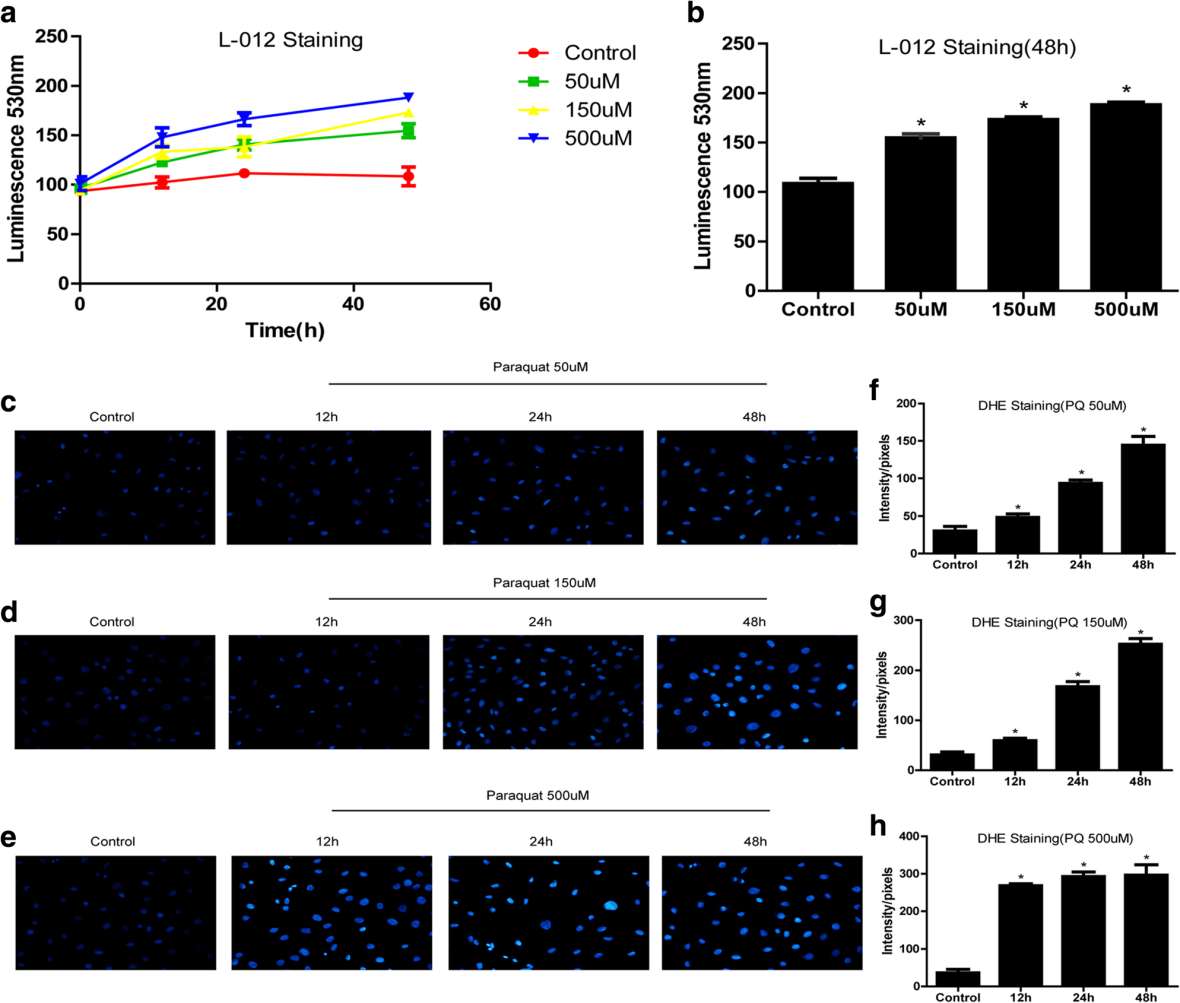
PQ causes a rise in intracellular ROS level and increased cell ROS generation. **a** 16HBE cells were subjected to different doses of PQ (50 μM, 150 μM, and 500 μM) at 12 h, 24 h, and 48 h, respectively, and the superoxide release was monitored by chemiluminescence dye L-012. **b** The luminescence values of L-012 dye in 16HBE cells treated with PQ (50 μM, 150 μM and, 500 μM) for 48 h. **c**, **d**, **e** Images and **f**, **g**, **h** quantification of dihydroethidium (DHE) staining for intracellular ROS by ImageJ software, the original objective magnification is × 40. Each assay in the experiments had at least 3 repetitions (the data are presented as mean ± SD, “*” means statistical significance).

According to the previous studies, ROS generation was pivotal for PQ-induced cell intoxication and lung fibrosis [7, 21]. Given the fact that ROS generation induces cell death and apoptosis [22], we next explored the extracellular superoxide release and ROS production in 16HBE cells treated with PQ. After treating 16HBE cells with PQ (50 μM, 150 μM, and 500 μM) for 48 h, L-012 assay results showed that extracellular superoxide production was increased by PQ treatments in a dose-dependent manner (Fig. 2a–b). The DHE staining data also showed the similar results; low doses (50 μM and 150 μM) of PQ slightly increase ROS levels at 12 h and 24 h, while high dose (500 μM) of PQ significantly increases ROS levels at 12 h (Fig. 2c–h), which indicated that PQ also induces ROS production in a dose-dependent manner, and our results were in accordance with the previous study [20].

### Cell Autophagy Was Affected in 16HBE Cells Co-Cultured with Different Doses of PQ

**Fig. 3:**
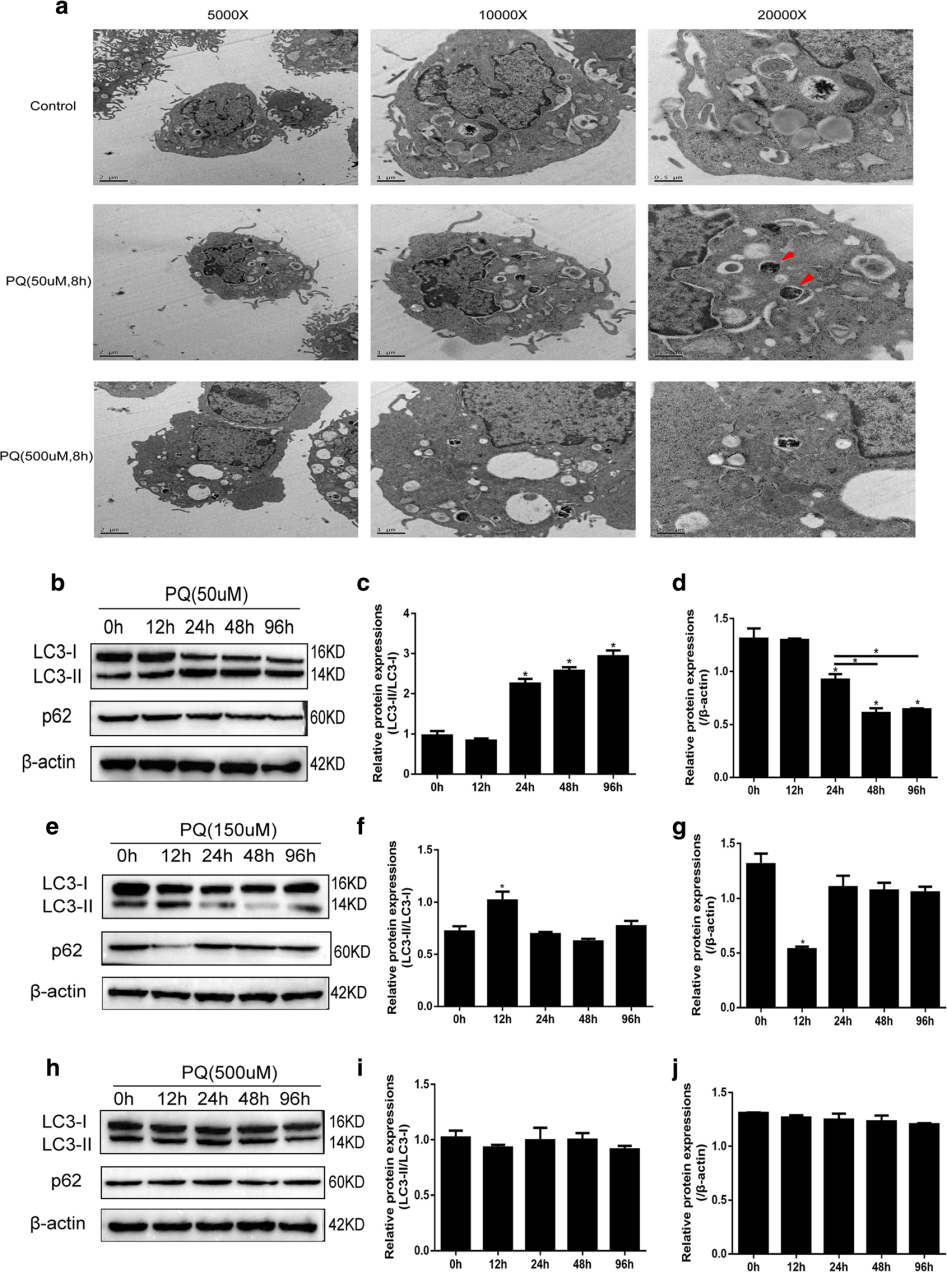
Different doses of PQ (50 μM, 150 μM, and 500 μM) had various impacts on cell autophagy. **a** Electronic microscope was used to observed cell autophagosomes in 16HBE cells treated with PQ (50 μM and 500 μM) for 8 h, the red arrows indicate the autophagosomes. **b**, **e**, **h** Western Blot was employed to detect cell autophagy-associated proteins in 16HBE cells treated with PQ (50 μM, 150 μM, and 500 μM) for 0 h, 12 h, 24 h, 48 h, and 96 h respectively. **c**, **d**, **f**, **g**, **i**, **j** Quantification of autophagy-associated proteins was conducted by ImageJ software according to the Western Blot results. Each assay in the experiments had at least 3 repetitions (the data are presented as mean ±SD, “*” means statistical significance, *p* < 0.05).

Cell autophagy has been reported to be closely related with PQ-induced cell intoxication [6]; since autophagy has dual effects on cells’ responses to environmental stress including ROS generation [23, 24], we next explored PQ’s influences on cell autophagy by electronic microscope and Western Blot. 16HBE cells were treated with different doses of PQ (50 μM and 500 μM) for 8 h; the images of electronic microscope showed that low dose of PQ (50 μM) induces autophagosome production in 16HBE cells, and the number of autophagosome dramatically decreased in 16HBE cells by treating cells with 500 μM of PQ (Fig. 3a). 16HBE cells were next treated with different doses of PQ (50 μM, 150 μM, and 500 μM) for 0 h, 12 h, 24 h, 48 h, and 96 h respectively; Western Blot results showed that 50 μM of PQ significantly increases LC3-II/LC3-I ratios and decreases p62 levels at 24 h, 48 h, and 96 h compared with the control group (Fig. 3b–d). Notably, compared with the control group, 150 μM of PQ merely increases LC3-II/LC3-I ratios at 12 h, which significantly decreases at 24 h, 48 h, and 96 h (Fig. 3e–f); p62 is also downregulated by 150 μM of PQ at 12 h (Fig. 3e, g). In addition, 500 μM of PQ has little impacts on either LC3-II/LC3-I ratios or p62 at any time point (Fig. 3h–j).

### PQ’s Influences on p65 and the Activation of Keap1/Nrf2 Signal Pathway

**Fig. 4:**
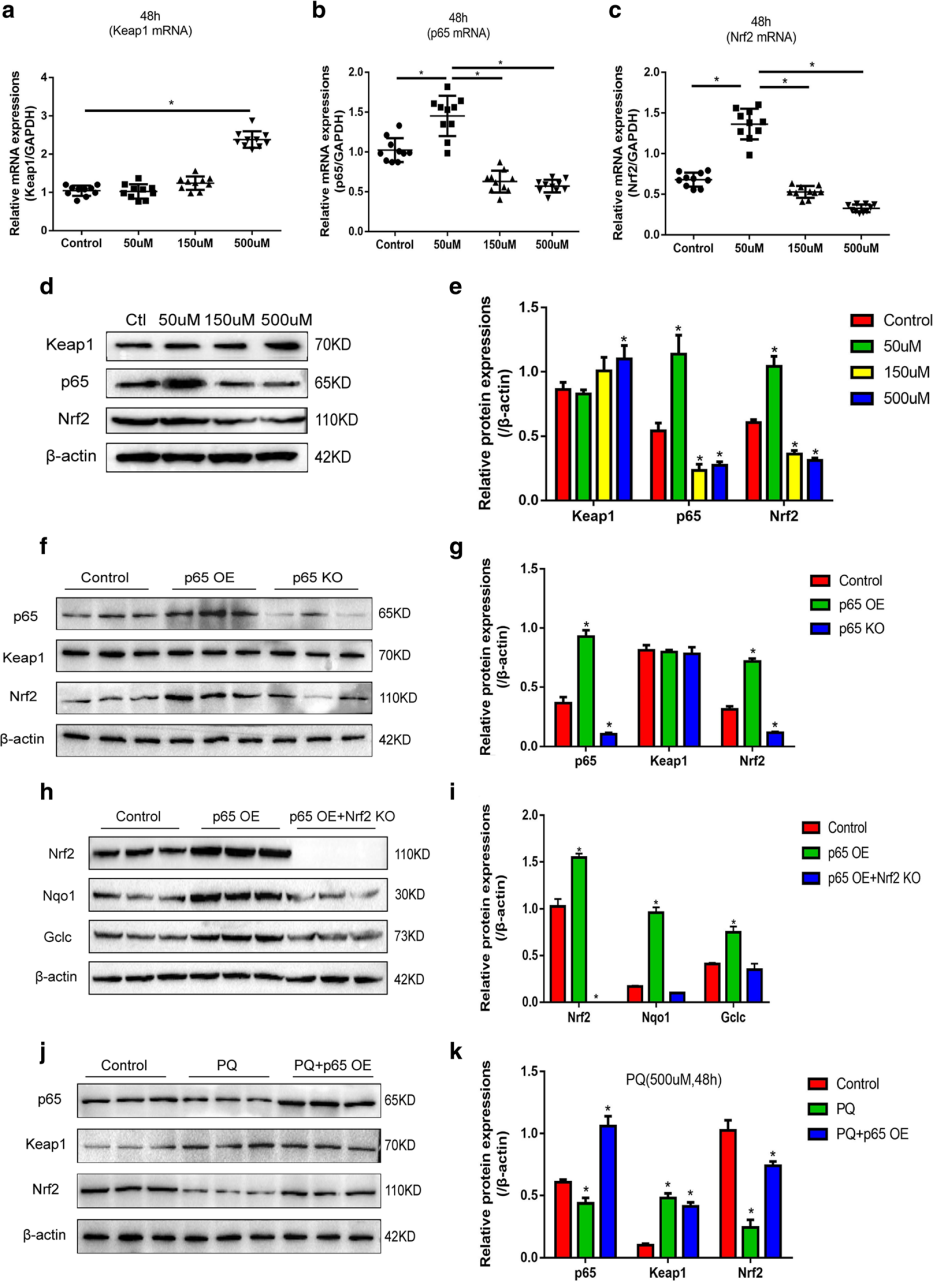
PQ’s influences on p65 and the activation of Keap1/Nrf2 signal pathway. **a**, **b**, **c** Real-time qPCR quantification of Keap1, p65, Nrf2 expressions, and normalized by GAPDH, each assay had 10 repetitions. **d** Western Blot was used to verify the expressions of Keap1, p65, and Nrf2 at protein levels. **e** Quantification of Keap1, p65, and Nrf2 by ImageJ software according to (**d**). **f** p65’s impacts on the expressions of Keap1 and Nrf2 were detected by Western Blot and **g** quantified by ImageJ software. **h** p65’s effects on Nrf2 downstream targets were detected by Western Blot and **i** quantified by ImageJ software. **j** High dose of PQ’s (500 μM) effects on p65, Keap1, and Nrf2 were detected by Western Blot and **k** quantified by ImageJ software. Each assay had 3 repetitions (the data are presented as mean ±SD, “*” means statistical significance, *p* < 0.05).

Our results showed that ROS generation plays an important role in PQ-induced cell intoxication, since Keap1/Nrf2 signal pathway is closely related with the regulation of ROS production [25], and p65 might influence Nrf2 by binding to its inhibitor Keap1 [17]. We next investigated the involvement of p65 and Keap1/Nrf2 signal pathway in 16HBE cells treated with PQ. 16HBE cells were treated with various doses of PQ (50 μM, 150 μM, and 500 μM) for 48 h; real-time qPCR results showed that 50 μM and 150 μM of PQ has little effects on Keap1 expressions compared with the control group, but Keap1 mRNA expression levels are significantly increased by treating 16HBE cells with 500 μM of PQ (Fig. 4a). Notably, 50 μM of PQ significantly increases p65 and Nrf2 mRNA expressions, which are surprisingly decreased by high doses of PQ (150 μM and 500 μM) (Fig. 4b–c). Further Western Blot results also showed that Keap1 is merely increased by treating cells with 500 μM of PQ. Besides, p65 and Nrf2 are upregulated by 50 μM of PQ, which are downregulated by treating 16HBE cells with high doses of PQ (150 μM and 500 μM) (Fig. 4d–e). The results also showed that Keap1 is not affected by p65 overexpression or knock-down (Fig. 4f–g). However, Nrf2 is significantly upregulated by p65 overexpression and downregulated by p65 knock-down compared with the control group (Fig. 4f–g). In addition, overexpressed p65 promotes Nrf2 downstream targets Nqo1 and Gclc expressions, which are reversed by synergistically knocking down Nrf2 (Fig. 4h–i). Furthermore, overexpressed p65 reverses 500 μM of PQ’s inhibiting effects on Nrf2 (Fig. 4h–i).

### Involvement of Keap1/p65/Nrf2 Signal Pathway in PQ-Induced Cell Intoxication

**Fig. 5:**
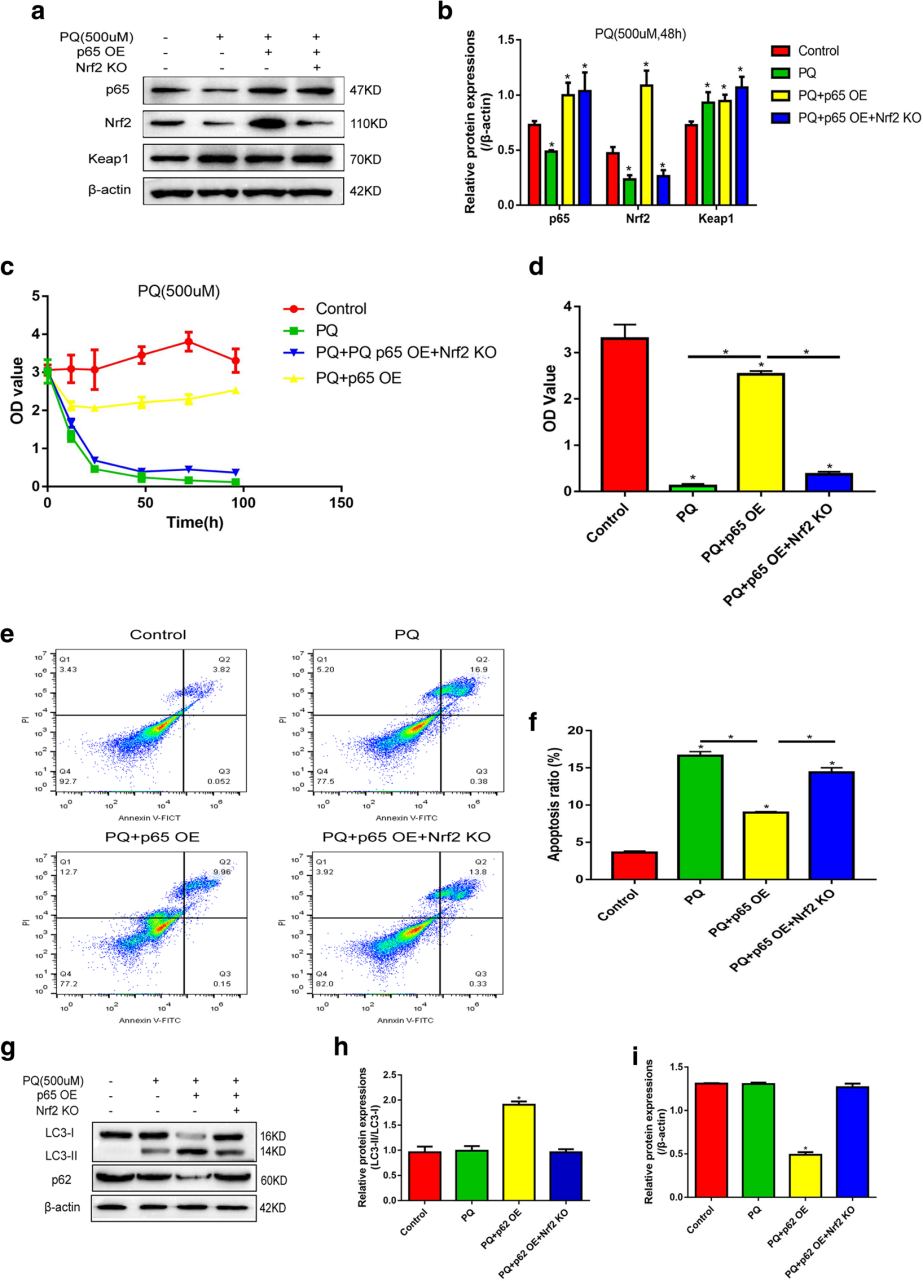
PQ regulates cell proliferation, cell death, and autophagy by modulating Keap1/p65/Nrf2 signal pathway. **a**, **b** Western Blot was used to verify the efficiency of p65 overexpression and Nrf2 knock-down in 16HBE cells. **c**, **d** The proliferative ability of 16HBE cells treated with 500 μM of PQ for 12 h, 24 h, 48 h, 72 h, and 96 h was detected by CCK-8 assay. **e**, **f** The apoptosis ratio of 16HBE cells treated with 150 μM PQ for 2 h was detected by FCM. **g**–**i** The autophagy-associated proteins of 16HBE cells treated with 500 μM of PQ for 48 h were detected by Western Blot. Each assay had 3 repetitions (the data are presented as mean ± SD, “*” means statistical significance, *p* < 0.05).

To explore the role of Keap1/p65/Nrf2 signal pathway in PQ-induced cell intoxication, we first overexpressed p65 or synergistically overexpressed p65 and knocked down Nrf2 in 16HBE cells (Fig. 5a–b). By treating cells with 500 μM of PQ for 12 h, 24 h, 48 h, 72 h, and 96 h, we found that cell proliferation is significantly inhibited by PQ in a time-dependent manner (Fig. 5c). Of note, overexpressed p65 alleviates PQ’s inhibiting effects on cell proliferation, which are reversed by synergistically knocking down Nrf2 (Fig. 5c–d). The FCM results showed that PQ (150 μM, 2 h) apparently increases 16HBE apoptosis ratio (Fig. 5e). Similarly, overexpressed p65 attenuates cell apoptosis, which is reversed by synergistically knocking down Nrf2 (Fig. 5e–f). In addition, compared with the control group and PQ-alone-treated group (500 μM), overexpressed p65 increases LC3-II/I ratio and decreases p62, which are also reversed by synergistically knocking down Nrf2 (Fig. 5g–i).

### Verification of Keap1/p65/Nrf2 Signal Pathway in PQ-Induced Acute Lung Intoxication by *In Vivo* Experiments

**Fig. 6:**
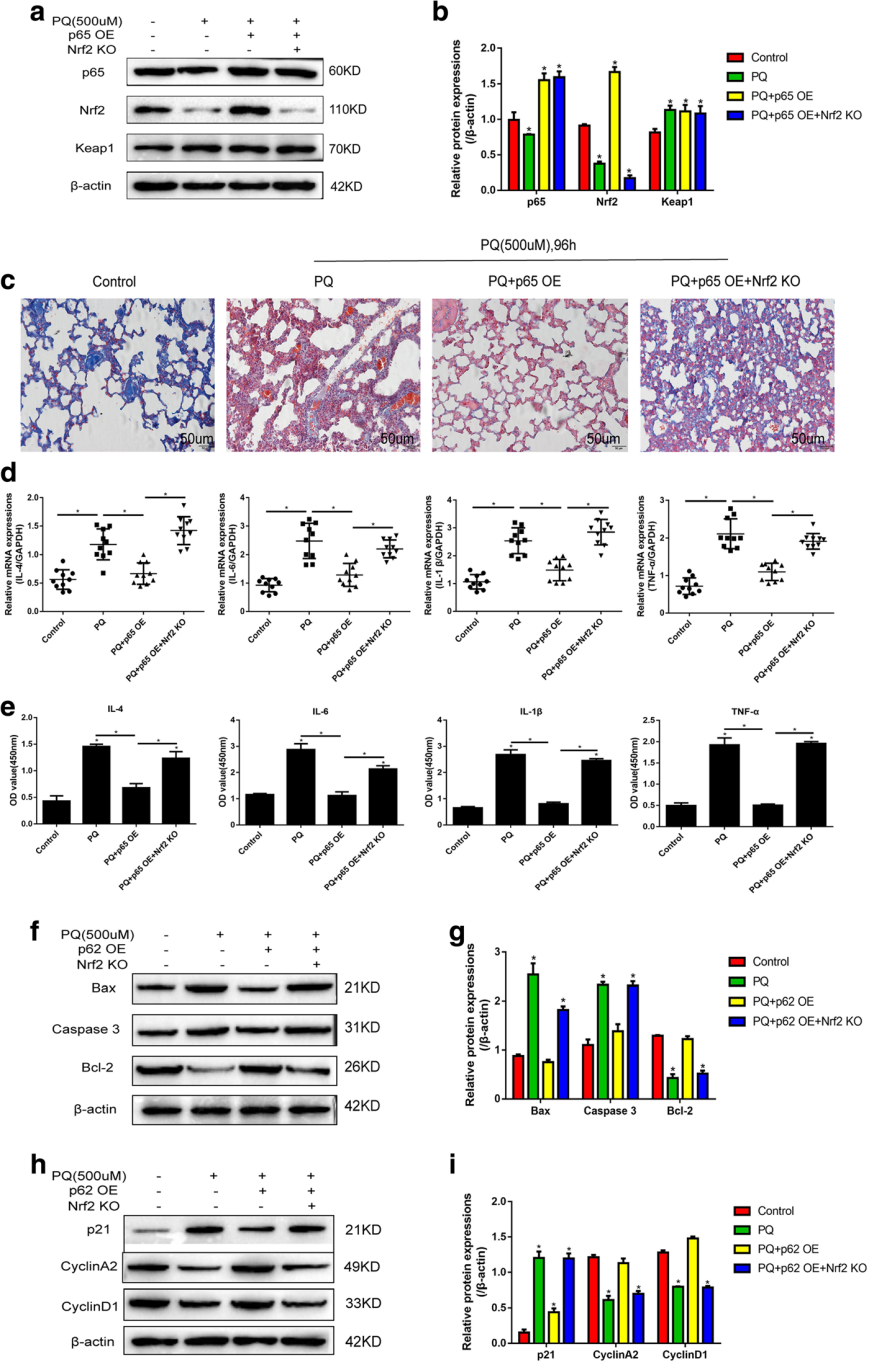
*In vivo* experiments prove that PQ induced cell intoxication by regulating Keap1/p65/Nrf2 signal pathway. Wild-type C57BL/6 male mice were intraperitoneal injected with saline or 500 μM of PQ and euthanized after 96 h. **a**, **b** Western Blot was used to verify and quantify the efficiency of p65 overexpression and Nrf2 knock-out in the lung tissues of the male C57BL/6 mice. **c** Masson staining was employed to observe the morphologies of the lung tissues of mice treated with PQ (500 μM, 96 h). **d** The relative mRNA expressions of inflammatory cytokines were detected by real-time qPCR in mice lung tissues and normalized by GAPDH. **e** The expressions of inflammatory cytokines in the periphery blood of the mice were detected by ELISA. **f**, **g** Cell apoptosis-associated proteins were detected and quantified in the mice lung tissues. **h**, **i** Cell cycle-associated proteins were detected and quantified in the mice lung tissues. Each assay had at least 3 repetitions (the data are presented as mean ± SD, “*” means statistical significance, *p* < 0.05).

To investigate the involvement of Keap1/p65/Nrf2 signal pathway activation in PQ-induced cell intoxication and lung fibrosis by *in vivo* experiments, male C57BL/6 mice were administered with 500 μM of PQ for 96 h to establish PQ-induced lung injury mice models. We first verified that we have successfully overexpressed p65 and knocked down Nrf2 in mice models (Fig. 6a–b). Masson staining images showed that lung fibrosis is induced by high-dose PQ treatment. Overexpressed p65 alleviates PQ-induced tissue morphology destruction, which is reversed by synergistically knocking down Nrf2 (Fig. 6c). PQ-induced lung fibrosis has also been reported to be seriously aggravated by inflammatory reactions; to investigate the role of Keap1/p65/Nrf2 signal pathway in regulating inflammatory reactions, real-time qPCR was used to detect inflammatory cytokine mRNA expression levels in lung tissues and ELISA was employed to detect their expressions in mice periphery blood (Fig. 6d–e). The results showed that high dose of PQ increases IL-4, IL-6, IL-1β, and TNF-α expressions in both mice lung tissues and periphery blood (Fig. 6d–e). Similarly, overexpressed p65 decreases IL-4, IL-6, IL-1β, and TNF-α levels in mice, which are reversed by knocking down Nrf2 (Fig. 6d–e). In addition, we found that PQ increases Bax and caspase 3 decreases Bcl-2 in mice tissues. Overexpressed p65 reverses PQ’s effects on the apoptosis-associated proteins, which are abrogated by synergistically overexpressing Nrf2 (Fig. 6f–g). Furthermore, overexpressed p65 also decreases p21 and increases cyclin A2 as well as cyclin D1 in mice compared with the PQ-treated group, which are also reversed by knocking down Nrf2 (Fig. 6h–i).

## DISCUSSION

PQ poisoning is a serious problem for human beings because there are currently no effective therapies for its treatment [26]. Studies have proved that PQ preferentially accumulates in patients’ lung and induces acute lung injury by regulating ROS-mediated cell death and activating inflammatory reactions [11, 27]. ROS-mediated autophagy-induced cell death also played an important role in PQ-induced cell intoxication [11], but the mechanisms are still unclear. The activation of Keap1/Nrf2 signal pathway was demonstrated to regulate ROS generation and inflammatory reactions [12, 18, 19], and p65 was capable of binding Keap1 and potentially participated in the inactivation of Keap1/Nrf2 signal pathway [17]. However, it is still unknown whether PQ induced cell intoxication and inflammatory reactions by regulating p65 and Keap1/Nrf2 signal pathway, and the influences of p65 on Keap1/Nrf2 signal pathway still need to be elucidated.

In our study, we found that PQ inhibits cell proliferation and promotes cell apoptosis in a dose-dependent manner, which is in accordance with previous study [20]. Notably, our results showed that PQ preferentially induces necrosis and late apoptosis instead of early apoptosis, which indicated that PQ induces acute cell apoptosis and cell necrosis is another characteristic of PQ-induced cell death; previous study suggested that PQ mainly causes necrosis in human neuronal cells [28], which was in line with our results. Autophagy is also induced by PQ [11, 29]; we found that autophagy is mainly induced by low dose of PQ (50 μM) instead of high doses of PQ (150 μM and 500 μM). The results above suggested that PQ’s influences on cell proliferation, autophagy, and death vary according to its concentrations.

Since ROS generation is an important factor in PQ-induced cell intoxication [7, 21, 30], we next explored PQ’s impacts on ROS generation. The results showed that PQ induced ROS generation in 16HBE cells in a dose-dependent manner. The results are in line with the previous study which reported that PQ-induced neurodevelopmental toxicity by promoting ROS generation [7]. Since it has been reported that ROS generation was closely related with cell autophagy [31], apoptosis [32], and viability [33], therefore, based on our results, we speculated that the effects of different doses of PQ on 16HBE cells might be associated with ROS generation, but the detailed mechanisms need to be deciphered in the further studies.

The activation of Keap1/Nrf2 signal pathway alleviates ROS generation by regulating AREs [16]; hence, we hypothesized that this signal pathway might participate in the processes of PQ-induced cell intoxication. Our results showed that this signal pathway is merely activated by low dose of PQ instead of high dose of PQ. In addition, Nrf2 is increased by p65 overexpression and decreased by p65 knock-down. Besides, the downstream targets (Nqo1 and Gclc) of Nrf2 are increased by p65 overexpression. Furthermore, overexpressed p65 also reverses high-dose PQ’s inhibiting effects on Nrf2 levels, which indicated that p65 promotes the release of Nrf2 and participates in the activation of Keap1/Nrf2 signal pathway.

Overexpressed p65 also decreases cell apoptosis ratio in 16HBE cells treated with high dose of PQ, which is reversed by synergistically knocking down Nrf2. Interestingly, high-dose PQ’s inhibiting effects on cell autophagy and proliferation are recovered by p65 overexpression, and synergistically knocking down Nrf2 abrogates p65’s promoting effects on cell autophagy and proliferation. The results above indicated that PQ modulates cell proliferation, death, and autophagy by regulating Keap1/p65/Nrf2 signal pathway. The *in vivo* experiments also verified that PQ induces mice lung fibrosis, inhibits cell proliferation, and promotes cell death and inflammatory cytokine secretion by regulating Keap1/p65/Nrf2 signal pathway. The results indicated that PQ triggers acute lung intoxication in mice by regulating Keap1/p65/Nrf2 signal pathway.

In conclusion, we found that high dose of PQ inhibits cell viability and autophagy and promotes cell death as well as mice lung fibrosis by regulating Keap1/p65/Nrf2 signal pathway.
